# Deep learning for carotid Doppler spectra classification

**DOI:** 10.1007/s11517-026-03569-1

**Published:** 2026-04-14

**Authors:** Charita Bhikha, Kahesh Dhuness, Mathilda Mennen, Normé Jamieson-Luff, Ntobeko A. B. Ntusi, Richard Wheatley

**Affiliations:** 1 Manufacturing Cluster, Council for Scientific and Industrial Research (CSIR), Pretoria, South Africa; 2 Division of Cardiology, Department of Medicine, University of Cape Town and University of Cape Town/South African Medical Research Council Extramural Unit On Intersection of Noncommunicable Diseases and Infectious Diseases, Cape Town, South Africa

**Keywords:** Artery identification, Carotid Doppler ultrasound, Explainable AI, Spectral Doppler, Transfer learning

## Abstract

Cardiovascular disease (CVD) remains a global health challenge, with limited specialist access in low- and middle-income countries hindering early detection. Carotid Doppler ultrasound offers promise for screening in non-specialist settings. However, spectral Doppler lacks the anatomical context provided by duplex ultrasound, making it challenging to determine which carotid vessel is being assessed. This study focuses on accurately identifying signals from the common, internal, and external carotid arteries (CCA, ICA, and ECA) based solely on Doppler spectra. This forms a critical step for subsequent disease classification. A clinical study enrolled 398 participants who underwent bilateral carotid Doppler examination (198 healthy controls, 200 with CVD). Several classifiers were evaluated including i) five deep convolutional neural networks (CNN) utilizing transfer learning on spectral images, and ii) conventional machine learning classifiers applied to the maximum frequency envelope and extracted features. The best performing classifier was a CNN (GoogLeNet) which achieved a mean area under the curve (AUC) of 0.929, effectively distinguishing between carotid artery segments. It exhibited f1-scores of 0.830, 0.803 and 0.764 for the ICA, ECA and CCA, respectively. Explainable AI tools (GradCAM and LIME) provide intuitive visual insights into these predictions. This study addresses a previously unsolved problem of automated carotid artery segment identification using spectral Doppler waveforms alone. When incorporated into automated screening tools, this approach provides a low-cost, specialist-independent pathway for earlier CVD detection, particularly suited to resource-constrained environments.

**Graphical abstract:**

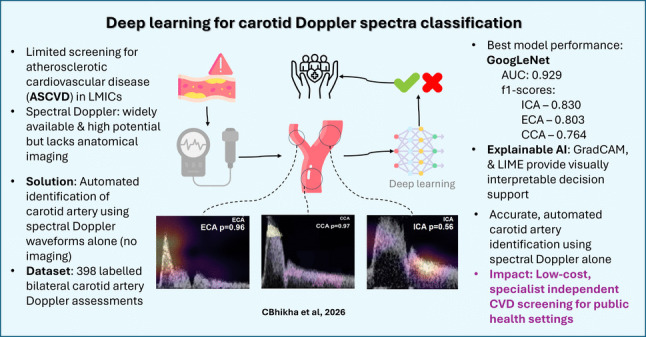

## Introduction

### Background

Noncommunicable diseases (NCDs) are a major global health challenge and the leading cause of death worldwide [1]. Among them, cardiovascular diseases (CVDs) account for 37% of NCD-related mortality [1]. The impact of NCDs is particularly severe in low- and middle-income countries (LMICs), where additional stressors such as poverty and a high burden of communicable diseases exacerbate the situation [2]. Recognizing the urgent need for action, the World Health Organization (WHO) introduced nine global targets in 2014 to prevent and control NCDs, including a 25% reduction in NCD mortality by 2025 [1]. A key component of achieving this goal is the early detection of CVD, which can help reduce its societal burden and improve patient outcomes.

Atherosclerotic cardiovascular disease (ASCVD), a major subset of CVD, is caused by atherosclerosis—a progressive condition in which plaque accumulates within the inner lining of blood vessels, restricting blood flow. Effective screening for ASCVD requires rapid, cost-effective, and non-invasive diagnostic tools [3]. Currently, coronary angiography is the gold standard for assessment, but it is costly, invasive, and necessitates a prior cardiac ultrasound for referral [3]. These procedures also require specialist expertise for both execution and interpretation, limiting their accessibility. As an alternative, Doppler ultrasound of the carotid artery offers a promising solution. This standalone technique is non-invasive, low-cost, and radiation-free, making it well-suited for widespread implementation in resource-limited settings [3].

Traditional duplex ultrasound systems combine imaging ultrasound with Doppler ultrasound capability. However, these systems are very costly and require specialist training. Spectral Doppler ultrasound refers to systems which produce only a Doppler signal as an output and do not have spatial imaging capabilities. They are also called vascular Doppler systems, commonly used to assess blood flow in the limbs for deep vein thrombosis (DVT) and peripheral arterial disease (PAD) [5]. They are also commonly used to monitor the umbilical artery during pregnancy [6]. The utility of spectral Doppler systems in primary healthcare settings has been shown to be effective in the case of monitoring the umbilical artery in the 3rd trimester of pregnancy for high resistance index, which is indicative of placental insufficiency [6]. Widespread screening using spectral Doppler has been shown to reduce stillbirth rates by 43% in South African [7, 8] and LMIC studies [9]. The significant reduction in stillbirth rate underscores the suitability and critical importance of this technology in meeting the demands of resource-limited environments.

In resource-constrained settings, spectral Doppler ultrasound emerges as a more viable solution than anatomical imaging systems. It is significantly more cost-effective, making it accessible for large-scale use in low-resource settings. Its ease of use means that it can be operated without extensive specialist training, which is a critical advantage in areas with a shortage of trained medical professionals. The equipment required for spectral Doppler ultrasound is more portable and easier to maintain compared to more advanced imaging technologies, which often need regular calibration and sophisticated maintenance procedures.

In LMICs, primary healthcare clinics serve the majority of the population, making them a crucial point of care for early disease detection and prevention. Integrating carotid artery screening at the primary care level could encourage healthier lifestyles, facilitate early intervention, and ensure that high-risk individuals receive timely referrals to specialized care [4]. To maximize its impact, it is essential to adapt this technology for use by frontline healthcare workers, such as nurses and community health workers, thereby enhancing healthcare accessibility and quality in LMICs.

### Spectral Doppler system limitations

Spectral Doppler systems differ from conventional duplex ultrasound in that they use simple transducer pairs without beamforming, insonifying the entire arterial cross-section and potentially introducing artefacts. The lack of imaging makes it impossible to determine the angle of insonation, preventing accurate velocity measurements like the Peak Systolic Velocity (PSV) and End-diastolic Velocity (EDV). Despite these limitations, relative Doppler indices such as Resistance Index (RI), Pulsatility Index (PI), and Systolic to Diastolic ratio (S/D) can still be calculated, though structural measurements like IMT and plaque size are unavailable.

### Carotid artery Doppler

The internal carotid artery (ICA), supplying the cerebral vasculature, is particularly important for evaluation, as plaque rupture and embolization can lead to stroke. Anatomically, the ICA is found posterolateral while the external carotid artery (ECA) is found more anteriorly and medially [10]. The ECA can be confirmed by using a ‘temporal tap’ which produces a serration like effect on the signal [10].

In traditional imaging ultrasound, the common carotid artery (CCA) is typically visualized near the base of the neck and then followed upwards, allowing visualization of the carotid bifurcation, the point where the artery splits into the ICA and ECA [10]. The bifurcation is an important site for evaluation of atherosclerosis, since it can have complex flow dynamics [11, 12]. This visual confirmation facilitates the identification of each segment and eliminates confusion with other vessels such as the major veins and smaller artery branches. With spectral Doppler, this approach is not possible, and identification of these arteries hinges solely on the operator’s skill, making use of anatomical landmarks and ability to recognize distinct waveform patterns of each segment. This subjective approach is inherently unreliable, particularly for non-specialists in sonography.

Therefore, prior to analyzing a signal for disease presence, it is necessary to first correctly identify the arterial segment, as each segment will have a characteristic normal appearance and set of parameters. There also exists a range of intra- and inter-individual variation due to anatomical variation, vessel tortuosity, disease presence, compensatory mechanisms due to disease [13], age, operator competence and measurement variation. These aspects of inherent variability and uncertainty pose a significant challenge in spectral Doppler analysis. Employing computational methods to automatically identify the segment of the carotid artery holds potential to enhance the reliability, objectivity, and consistency of spectral Doppler carotid artery screening.

### Prior work

Prior work on AI application to carotid Doppler ultrasound spectra make use of extracted features, such as the maximum frequency envelope [14–17], power spectral density of the entire waveform [18], absolute velocity values or manual descriptions of the waveform [19], and mostly aim to predict disease, since the waveform origin is already known to be the ICA. In terms of deep learning application to Doppler images, one study employed transfer learning on multiple CNNs to classify Doppler images of mitral valve inflow into 24 classes with an overall accuracy of 0.97 [20].

Some evidence suggests that Doppler indices such as the RI may aid in distinguishing between different carotid arteries [21]. However, substantial variability and overlap in the range of normal values, make threshold-based cut-off values insufficiently accurate [18]. Furthermore, disease states can significantly alter the Doppler waveform’s appearance and associated parameters.

To the best of our knowledge, no research has been conducted to distinguish between carotid artery segments using computational methods applied to Doppler spectra. This lack of prior work can likely be attributed to the fact that differentiation between the segments is usually not necessary in routine clinical examinations. Trained sonographers or clinicians typically perform the examination using phased array imaging systems, relying on their anatomical knowledge, recognition of typical signal patterns, and established techniques to visualize the segments. Furthermore, standard carotid evaluation involves imaging of the artery and plaque characterization, rather than relying solely on Doppler measurements. However, with the intention of using spectral Doppler alone to screen for CVD, the carotid artery segment must first be accurately identified before analysis for CVD can take place.

### Contribution of this study

This work presents a novel framework for applying machine and deep learning methods to carotid Doppler spectra from a cohort of healthy participants and patients with established CVD. The aim is to classify carotid Doppler spectra by their origin of ICA, ECA or CCA, which is an important precursor to subsequent analysis for potential abnormalities. This would overcome a significant hurdle in applying spectral Doppler alone for the detection of CVD in non-specialty environments. Several machine and deep learning models are compared and evaluated, and insight into class features are gained through explainable AI tools. The framework presented is suitable for application to Doppler ultrasound spectra in general.

## Methods

### Data collection

This work is a retrospective analysis of data collected during a cross-sectional study conducted between August 2021 and March 2023. The study was performed at the Department of Medicine, University of Cape Town at Groote Schuur Hospital and the Cape Universities Body Imaging Centre (CUBIC) in South Africa. The study cohort comprised 198 healthy adult controls and 200 patients diagnosed with established cardiovascular disease (CVD), or other cardiovascular conditions based on pre-defined inclusion criteria. The CVD cohort comprised patients with confirmed cardiovascular conditions (e.g., ischemic heart disease, heart failure, or peripheral vascular disease), irrespective of the presence of carotid plaque. All procedures adhered to relevant laws and institutional guidelines, with ethics approval granted by the University of Cape Town and CSIR Ethics boards (UCT HREC REF: 749/2020 and CSIR REC registration number: 383/2021). The privacy rights of participants have been observed and informed consent was obtained.

Data acquisition was performed using a GE Vivid E95 ultrasound system equipped with a 4 MHz phased array probe. The imaging modality provided the anatomical ground truth to identify the ICA, ECA and CCA. All participants underwent a comprehensive bilateral carotid artery ultrasound examination of the common, internal, and external carotid segments. This yielded six Doppler waveforms per participant. In addition to acquiring waveform data, the peak systolic velocity (PSV), end diastolic velocity (EDV), intima-media thickness (IMT) and lumen diameter were measured for each carotid artery segment. An experienced sonographer conducted the examinations and performed data labeling.

Duplex ultrasound from an imaging machine was used as a reference to assess the performance of the classification models, given that anatomical imaging is unavailable in spectral Doppler systems. The intention is to then apply these models to a spectral Doppler system.

Incorporating participants that are both healthy and with cardiovascular conditions into model development is crucial for achieving generalizability. As shown in Table 1, significant statistical differences exist between the two groups in age, sex, systolic blood pressure, respiratory rate, lifestyle factors, and medical history. These factors can influence Doppler waveform presentation due to variations in cardiovascular function and hemodynamics. Including both groups allows the model to account for this inherent variability and enhance its ability to perform accurately across a broader population.

**Table 1:** Demographic and clinical characteristics of study participants

		All	Control	CVD	*p*-value (*0 = < 0.0001)
Samples	n	398	198	200	0.5
Female: Male	n	230:169	140:59	90:110	0:0
Ethnicity	n [B:A:M:W]	59:4:268:65	50:2:114:32	9:2:154:33	0:0.5:0:0.46
Age	mean (range)	55 (22–86)	46 (23–76)	63 (22–86)	0
Risk factors					
Blood pressure	mean (SD)	139/82 (20.27/12.22)	134/81 (19.47/11.77)	144/84 (19.97/12.47)	0/0.007
Heart rate	mean (SD)	80 (33.47)	83.50 (44.96)	76.40 (13.91)	0.0178
BMI	mean (SD)	30.14 (8.14)	30.71 (8.6)	29.56 (7.61)	0.0792
Respiratory rate	mean (SD)	13.83 (2.74)	12.77 (1.57)	14.90 (3.21)	0
Smoking	n	145	45	100	0
Family history CVD	n	222	116	107	0.1536
Cardiac disorder	n	189	35	154	0
Hypertension	n	99	14	85	0
Hyperlipidemia	n	93	20	74	0
Statin Therapy	n	139	15	125	0

*SD* Standard deviation; [B:A:M:W] = [black,asian,mixed,white]

### Data processing

All computational analyses were conducted within the MATLAB R2024a environment. Processing was performed on a laptop equipped with an Intel Core i7-13850HX processor, 32GB of RAM, and an NVIDIA RTX 3500 ADA GPU.

Following data acquisition with the GE Vivid E95 ultrasound system, the data is exported in DICOM format. Doppler spectral images are then extracted using predetermined pixel coordinates. Textual data, including PSV, EDV, and IMT values, is obtained via optical character recognition (OCR). In contrast, a spectral Doppler-only system typically outputs the signal as a two-channel audio stream containing quadrature signals shifted by 90 degrees. These signals can be processed to generate forward and reverse Doppler spectral images by applying a complex short-time Fourier transform (STFT) [29] with a sliding window size ranging from 256 to 1024 values.

**Fig. 1:**
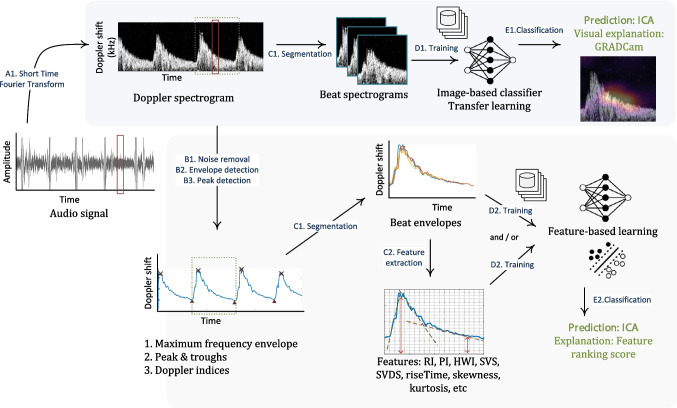
The data processing pathway for a signal from a Doppler probe through to a classification result. The upper panel shows the processing pipeline for deep learning application to spectral images while the lower panel illustrates the processing pathway followed for conventional machine learning analysis on the spectral envelope and derived features

To simulate real-world spectral Doppler systems, this model solely relies on the Doppler spectral images and features extracted from them. Absolute velocity and spatial measurements, while potentially informative, are excluded as inputs as they wouldn’t be obtainable in a typical spectral Doppler system. Figure 1 illustrates the various preprocessing stages, which are further detailed in the following text.

#### Noise reduction

The dataset contained samples of varying signal strengths and signal-to-noise ratios (SNR). Histogram equalization was applied to create consistent signal strength across the samples. These steps avoid accuracy loss due to poor sample quality and prevents erroneous learning of noise or signal strength as a characteristic feature, allowing the classifier to be applied to samples from various sources with varying SNR. Noise removal is also crucial to ensure that a well-defined envelope can be extracted.

#### Envelope detection

The maximum frequency envelope of the signals is obtained using an image processing technique, which was found to be more robust than existing methods for the dataset at hand. Methods like the geometric method [22] and signal–noise slope intersection [23] make use of the cumulative power distribution function for each column of data. These methods were found to be sensitive to SNR and capture spurious values. The image processing technique used involves using morphological dilation and erosion to create a consistent signal region without holes and gaps, removing noise and maintaining details of the envelope shape. The envelope is then extracted from the boundary of the largest segmented component in the image.

#### Peak detection

The peaks and troughs are obtained by searching for all local maxima and minima on the envelope. The peaks are reduced such that they have a minimum distance between them which relates to a maximum heart rate of 220 beats per minute. The peaks are also filtered to ensure a consistent mean height, eliminating outliers. Adjacent peaks and troughs are also removed. This ensures a final set of consistent alternating peaks and troughs.

#### Doppler indices

Traditional Doppler indices of the RI, PI, S/D and heart rate can be calculated once the peaks and troughs have been obtained. For an envelope y:

$$\begin{array}{l}SD=\mathrm{max}(y)/\mathrm{min}(y)\\ RI=(\mathrm{max}(y)-\mathrm{min}(y))/\mathrm{max}(y)\\ PI=(\mathrm{max}(y)-\mathrm{min}(y))/\mathrm{mean}(y)\end{array}$$

#### Segmentation

The troughs are used to segment the envelope and spectra into sections corresponding to cardiac cycles, or heart beats. A window whose width relates to the heart rate can also be used to extract the beats, however this can be less accurate especially if there is an arrythmia present. The segmented images are resized to a consistent size, and the envelope segments are also resampled such that all segments have the same length. An average of 3.5 useable beats are extracted from each sample, resulting in a total sample size of 8067 beats. Table 2 describes the subsets sizes per artery and category.

**Table 2:** Dataset sizes and categories. Six samples are acquired from each patient. Each sample contains an average of 3.5 beat segments. The table shows the number of samples and segments after removal of unusable samples

	Control	CVD	All
nPatients	198	200	398
ICA samples	373	411	784
ECA samples	353	418	771
CCA samples	374	399	773
Total samples	1100	1228	2328
No. Segments per sample—mean			3.5
ICA segments	1400	1401	2801
ECA segments	1324	1209	2533
CCA segments	1420	1313	2733
Total segments	4144	3923	**8067**

#### Normalization

The vertical height of the envelope is the Doppler shift, which is related to, but not equal to, the absolute blood flow velocity. The Doppler shift also varies with the Doppler angle, which is operator and measurement dependent. The effect due to this non-deterministic angle needs to be removed, which is done by normalizing the envelope. The following normalization processes are thus applied to the data to compensate for sources of variability:

1. Varying & unknown Doppler angle: Normalize vertical maximum to 1
2. Varying heart rate: Signal compression/expansion via segmentation and sample resizing
3. Cardiac cycle phase: Horizontal shift negated via segmentation by troughs
4. Noise: Filtering & spectral subtraction of background noise
5. Signal strength: Normalize spectral intensity via histogram equalization and intensity normalization

#### Feature extraction

A total of 23 additional features are derived by further processing each segmented Doppler envelope. For each segment, systolic and diastolic phases are identified using the midpoint on the descending slope between the systolic peak velocity and end-diastolic velocity. Based on these phase definitions, various slope gradients, time intervals, ratios, area and shape-based descriptors are then calculated to form the following extracted features:

1. Resistance index
2. Pulsatility index
3. Systolic to diastolic velocity ratio
4. Pulse duration
5. Maximum Doppler shift
6. Systolic velocity upward slope/acceleration
7. Systolic velocity downward slope
8. Diastolic velocity slope
9. Slope from base to peak
10.  Systolic rise-time
11.  Systolic duration
12.  Systolic peak duration
13.  Systolic percentage
14.  Diastolic duration
15.  Peak top 10% duration
16.  SD duration ratio
17.  Area under curve
18.  Area under peak
19.  Height-width index (HWI)
20.  Modified Height-width index (HWI2)
21.  Skewness
22.  Kurtosis
23.  Number of peaks

### Learning approaches

The original Doppler spectral images are processed and segmented into beats as outlined, increasing the dataset approximately fourfold. This pre-processed dataset is then categorized into sets of ICA, ECA and CCA. Each of these groups are then split randomly into training, validation and test sets using a ratio of 7:2:1 respectively, ensuring each set has a balanced ratio of samples from each artery type. However, all beat segments originating from the same waveform are kept together, so that samples across the training, validation and test sets do not contain the same or similar samples (as this would defeat the purpose of the validation and test sets). Patient-level splitting is also enforced to eliminate potential statistical dependance between samples originating from the same individual. The data sets are kept constant such that the results across multiple classifiers can be compared in a fair manner. However, a number of these randomly generated datasets are created to ensure model reproducibility.

Both machine and deep learning methods are considered. The aim of applying various models is to compare performance, find an appropriate model size to match the complexity of the problem, to evaluate the usefulness of various feature sets and to explore the insights offered by each.

To ensure generalization and avoid overfitting, cross-validation is used. The test set is expanded by the addition of augmented test samples (randomly applying maximum ± 2 degrees rotation, ± 10 pixel horizontal and vertical translation and 0.9–1.1 factor scaling). All models are evaluated using the Receiver-Operating-Curve (ROC) and the area under the curve (AUC), as well as elements of the confusion matrix (sensitivity, specificity, and f1-score) on the unseen test dataset. When referring to model ‘performance’, reference is being made to these parameters. The f1-score is chosen as the main performance parameter as it combines precision and sensitivity, both of which need to be high for a good model.

#### Conventional machine learning methods

Various machine learning classifiers are considered including random forest, k-nearest neighbor, SVM, and 3-layer neural network. They are applied to i) the segmented maximum frequency envelopes and ii) 23 extracted features from the envelope segments. The envelope detection algorithm and feature extraction stages need to be robust to produce good quality feature sets; any inaccuracies introduce an error margin into the data. Excessively noisy samples and outliers are removed to improve data quality. The exclusion criteria include samples where pre-processing has failed or where extracted features are more than three standard deviations away from the mean. To analyze the impact of the various features on overall classification, the Chi-squared feature ranking method is used.

#### Deep learning methods

Deep learning neural networks are used to analyze the spectral beat images directly, having the inherent advantage of automated feature extraction. Designing an appropriate neural network architecture can be a complex task, with a wealth of different layer types available. To ensure that a well-designed architecture is used, several well-known deep learning models are applied using transfer learning. These include GoogLeNet (Inceptionv1)[24], EfficientNet-b0, DenseNet201, ResNet50, AlexNet and VGG16, which were all originally well-trained on the ImageNet database of 1.2 million images [24]. Transfer learning is the process of using a pre-trained model, usually a neural network, as a starting point for training on new data. It is beneficial as it allows the model to retain important abilities learned from a large prior dataset (for example low-level features such as edges and textures) and learn new high-level features from a smaller custom dataset. This approach of comparing several well-known transfer learning models has become common, for example the approach has been applied to carotid artery plaque classification [25], the detection of monkeypox skin lesions [26] and the classification of lung CT scan for COVID19 detection [27].

The final convolutional layer of each network is replaced by a new fully connected layer to obtain the output for the three artery classes. Spectral beat images are converted from grayscale to color (RGB) using a colormap index and resized to match the network’s expected input size. Noisy samples are not excluded from the dataset to allow models to generalize across signals of various SNRs. During training, the weights of both the pretrained layers and the new layer of the network are fine-tuned at the same rate using the Stochastic gradient descent with momentum (SGDM) optimizer. Optimal parameters include a learning rate of 1e-4, a mini-batch size of 32, and a regularization parameter of 0.001, which are used across all final models. This configuration is found to produce stable convergence while minimizing overfitting.

Cross-validation is employed to halt training once a steady state is achieved. When the validation and training set loss graphs are seen to diverge, training is stopped, i.e. the training loss continues to decrease while the validation loss remains relatively constant. This indicates that the model is starting to overfit the training data. It is known that a network of sufficient complexity will always learn the training set perfectly including all fine detail such as any noise [18]. The weights from the best validation accuracy run are taken as the output. The models train for an average of 3–4 epochs.

#### Explanation tools

To aid explanation on the behavior of a model and gain insight into the results, Gradient-weighted Class Activation Mapping (GradCAM) and Locally-Interpretable Model-agnostic Explanation (LIME) tools are used. GradCAM uses the activations in the last convolutional layer to highlight important input features [28]. LIME creates perturbations around local neighborhoods in the input sample and linearly models the effect of the perturbations on the model’s output [29], thereby determining the importance of features in the input. These tools are used to create a heatmap overlay on the image which conveys a visual explanation or justification for the predicted result, and is useful in seeing a pattern of importance both globally within in a class and locally on specific samples.

## Results

The prepared datasets are used to train and evaluate various learning models. All model results are evaluated on the test dataset consisting of previously unseen samples.

A summary of the performance of the models considered is presented in Table 3. The term ‘performance’ is used generally, referring to the overall combination of AUC, f1-score, sensitivity and specificity. Only the machine learning models with best performance for each unique input set are shown, even though many others of poorer performance were evaluated during the study.

**Table 3:** The performance of 1) threshold-based classifying; 2) Machine learning models with extracted envelope features as input; 3) Machine learning models with the maximum frequency envelope as input; 4) Deep learning-based models operating on images of the spectral beats as inputs. Models are ordered by increasing number of parameters

#	Model	# Parameters	# Layers	ICA			ECA			CCA		
				AUC	F1-score	Specificity	AUC	F1-score	Specificity	AUC	F1-score	Specificity
	**Threshold-based:**											
1.1	RI [0.70,0.77]	2		0.767	70.6	82	0.727	72.2	76.8	0.544	59.8	70.7
1.2	PI [1.00,1.35]	2		0.765	70.8	83.3	0.727	71.8	75.5	0.534	62.1	81.2
1.3	EDV [14, 25]cm/s	1		0.723	66.9	81.3	0.741	58	85.2	0.446	58.5	61.6
	**ML:**											
2.1	ML-rForestFeatures			0.845	68.8	75.1	0.870	68.1	91.1	0.896	75.9	90.2
2.2	ML-rForestRIPISD			0.758	55.6	67.4	0.778	55.5	60.2	0.711	56.3	53.3
3.0	ML-NN-envelope		3	0.799	65.7	78.8	0.807	67.5	89.6	0.837	71.1	83.6
	**DL-Spectra:**											
4.1	DL-efficientnet-b0	5.3 M	82	0.878	73.4	82.1	0.865	62.4	93.6	0.831	68.0	76.5
4.2	DL-googlenet	7.0 M	22	**0.948**	83.0	**92.9**	**0.945**	**80.3**	86.6	**0.894**	**76.4**	**90.7**
4.3	DL-densenet201	20.0 M	201	0.911	76.4	87.7	0.906	70.4	92.9	0.871	72.8	79.1
4.4	DL-resnet50	25.5 M	50	0.940	80.3	92.1	0.919	75.5	95.3	0.881	74.0	77.1
4.5	DL-alexnet	60.9 M	8	0.939	81.0	89.0	0.930	77.8	**95.5**	0.871	73.6	81.1
4.6	DL-vgg16	138.3 M	16	0.942	**83.8**	90.5	0.925	77.2	91.3	0.885	75.8	86.8

*ML* Machine learning; *DL* Deep learning; *NN* Neural network; *AUC* Area under ROC curve; *rForest* Random forest

### Comparison of classifiers

**Fig. 2:**
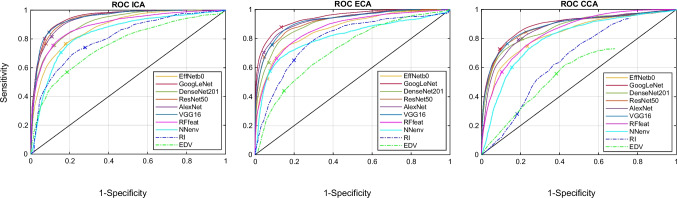
The ROC curves for all the deep learning models, and the top machine learning models for both the envelope and feature based input and the ROC for simple classification by RI and EDV thresholds

Table 3 presents the performance of progressively more complex classifiers, spanning traditional threshold-based indices, conventional machine learning (ML), and deep learning (DL) models. The corresponding ROC curves for the models are shown in Fig. 2. Generally, as the number of learning inputs increases, the AUC increases up to a point, where increased model complexity no longer results in further performance increases. Although the ICA classifier tends to show marginally higher AUCs, overall performance across arteries is comparable.

The models do not always operate at the optimal operating point on each class-specific ROC curve, as the nature of the multiclass classifier means all three operating points are linked. Rather, the operating points are determined by optimizing overall classification across all the classes.

The simple threshold-based methods using RI, PI, and EDV (rows 1.1—1.3 in Table 3) show modest discriminative ability, with AUC values generally below 0.77 and particularly weak performance for the CCA (AUC ≤ 0.54).

Using feature-based machine learning methods yields a clear improvement over thresholding. A random forest model operating on the set of 23 extracted envelope features (row 2.1 in Table 3) achieves substantially higher AUCs (0.845–0.896) and a mean f1-score of 71.0% across all arteries, demonstrating the advantage of curated features condensing known useful information. The model performing best on the raw maximum frequency envelope (tri-layered neural network, row 3.0 in Table 3) did not perform as well as the random forest in 2.1. Performance further degrades when the feature set is constrained to the 3 traditional Doppler indices (RI, PI, SD) (row 2.2 in Table 3).

The best overall performance is obtained with deep learning models applied directly to Doppler spectra, which consistently outperform ML models. Among these, the GoogLeNet CNN is the best performing model across all artery classes (mean f1-score of 79.9% and AUC of 0.929) and displayed the best performance for its size. The DeLong comparison test for AUCs between GoogleNet and VGG16, the second-best model, yielded p values of 0.42, 0.00062 and 0.0398 for the ICA, ECA and CCA respectively, demonstrating a significant difference in ECA and CCA classification. DenseNet201 and EffcientNet-b0 has the poorest performance amongst the deep learning models. While larger architectures such as VGG16, AlexNet and ResNet50 have high AUCs, they have non-optimal operating points (i.e. low specificities, or low sensitivities to maintain high specificities, see Fig. 2).

**Fig. 3:**
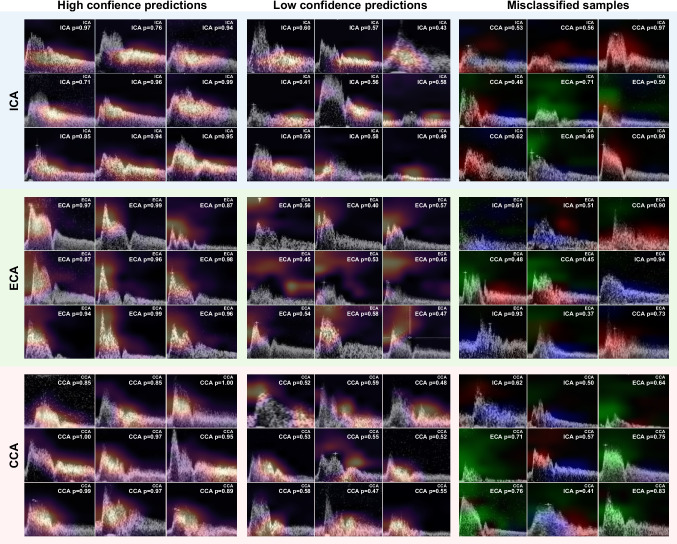
Classification output of the best performing CNN (GoogLeNet). In each image, the true label is shown on the top right with the predicted class and the probability below. Samples of the ICA (top panel), ECA (middle panel) and CCA (bottom panel) labelled groups showing GradCAM heatmap activations. Left column: high confidence correct predictions, Middle column: low confidence correct predictions, Right column: misclassified samples with colored feature activations shown to aid potential explanation for misclassifications (Red – CCA activations, Green – ECA activations, Blue – ICA activations.)

Figure 3 displays a sample set of input Doppler spectra, per artery class, together with the classification outputs and GradCAM overlays of the best performing classifier (GoogLeNet). A model’s prediction consists of a confidence score for each arterial class, with the scores summing to unity. The class with the highest confidence score is designated as the predicted outcome. As can be seen in Fig. 3, high model confidence (> 0.7) coincides with Doppler waveforms exhibiting the expected characteristics for the assigned class.

## Discussion

### Machine- versus deep learning approaches

The modest performance of the threshold-based RI, PI, and EDV methods highlights these parameters’ importance as features, but also shows their restricted capacity in capturing complex spectral information. Additional refined features are needed to reach higher accuracy. In the presence of CVD, these values are known to deviate away from the normal ranges and hence, they may be more useful in the disease classification problem.

The improved performance of conventional machine learning models demonstrates the value of multivariate feature integration. The condensed set of engineered features are well-suited to the random forest model where iterative decision making is made. Even though the ML models were exposed to the full raw envelope, they may not have had the complexity required to internally extract sufficiently complex features to outperform the explicitly engineered feature set. The drop in performance when constrained to only the 3 traditional Doppler indices (RI, PI, SD) (row 2.2 in Table 3), highlights the importance of the additional curated features, which more fully characterize the complete Doppler signal.

The random forest models are prone to overfitting the training data. Pruning and regularization improved generalization but decreased performance on validation and test sets. Even a slight change to samples in the training data resulted in a large performance drop. This may indicate that the machine learning models may not do well in the presence of noise or signals from a different source.

An advantage of the traditional feature-based machine learning approach is that models are less complex and hence computationally more efficient compared to deep learning models. This allows them to be easily deployed to low-resource computing platforms or edge devices. Condensing information into curated features that are known to be relevant could focus the model and reduce the chance of overfitting to noise and spurious features. The results are explainable via LIME and feature ranking methods, although this is not as intuitive as image-based methods especially to an end-user.

The results in Table 3 and Fig. 2 collectively demonstrate that end-to-end deep learning models leveraging full spectral information provide the most accurate and balanced classification, with overall higher F1-scores and AUCs. Differences in layer architecture and training parameters contribute to differences in performance among the deep learning models.

There are several advantages to the deep learning approach: by intrinsically extracting features that are statistically relevant, potentially important information is not discarded, especially those that are not easily human-perceptible. Because it operates on the spectra directly, there is no need for feature extraction algorithms such as envelope and peak detection, which can introduce error if not robust enough. Deep learning models can be trained on samples of varying SNR with high levels of background noise, where feature-based methods would fail due to inaccurate feature extraction itself. Furthermore, explanation of the results via GradCAM or LIME tools provide a visual insight which is engaging and intuitive. This would be increasingly valuable for the application of disease detection, where difficulty in understanding why certain prediction is made would make it challenging for the user to trust the model.

The impact of signal source variability on performance is multifaceted. Machine learning models might benefit by isolating the envelope and Doppler features, thereby potentially mitigating the influence of significant variations caused by factors like noise, SNR (signal-to-noise ratio), and sample volume discrepancies inherent to different measuring devices. However, this necessitates the development of robust pre-processing stages capable of handling diverse sample sources. Conversely, deep learning models may exhibit inherent robustness to noise and artifacts, potentially rendering them less susceptible to variations in sample source. However, significant signal differences might necessitate retraining on new data especially from spectral Doppler devices for optimal performance.

### Feature insight using explainable AI methods

To gain an understanding of model layers and gain insights into the features they utilize for classification, explainable AI methods are employed. The activation overlays produced by GradCAM and the LIME feature importance map offer useful insights into the unique characteristics of each artery segment.

#### GradCAM

GradCAM analysis utilized the class activation maps generated from the final fully connected layer. Among the CNN architectures, GoogLeNet produced the most intuitive results (Fig. 3). The highlighted regions show patterns most responsible for the prediction made, aligning with known vessel physiology. For the ICA (Fig. 3, top panel), predictions focused on prominent, slowly decreasing flow in the late systolic and early diastolic regions. This aligns with the ICA’s role in supplying a low-resistance downstream bed with consistent capillary parameters, resulting in continuous blood flow throughout the cardiac cycle. Conversely, the ECA group (Fig. 3 middle panel) exhibited strong activation in areas corresponding to the sharp triangular systolic peaks and the diastolic notch. These high-frequency features are characteristic of the ECA, which supplies highly dynamic capillary beds in facial structures. The ECA experiences greater fluctuations due to temperature and muscle activity, often exhibiting low or absent diastolic flow [30]. Finally, the CCA activations (Fig. 3 bottom panel) emphasized a broader, petal-shaped systolic pulse with the presence of both diastolic flow and a highlighted diastolic notch. This aligns with the CCA’s role, as it naturally integrates flow characteristics of both the ICA and ECA.

Distinguishing the ECA from the CCA can be challenging due to visual similarities. When a sample exhibits characteristics of multiple segments, the classification outcome result is weighted across the classes, resulting in a low to medium overall confidence score.

**Fig. 4:**
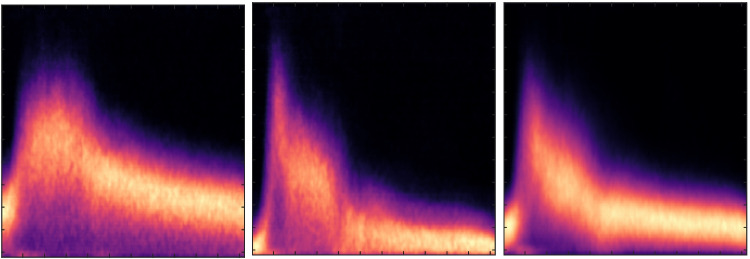
Averaged samples of the internal (left), external (middle) and common (right) carotid arteries illustrating their characteristic appearance

As the waveforms deviate from the expected appearance, the confidence score correspondingly decreases. To illustrate the arteries’ typical appearances, Fig. 4 presents the averages of high confidence predictions for each segment. Significant deviations from these conventional patterns might suggest outliers, mislabeled data, or severe vessel pathology. Misclassified samples, upon examination, often display features resembling the predicted class rather than the labelled class.

Samples with strong activations in multiple classes typically exhibit low confidence scores. Notably, samples with mixed features often display overlapping features of the CCA and either the ICA or ECA, as can be seen in the last column of misclassifications in Fig. 3. These could be cases of the measurement location being at the distal CCA or proximal ICA or ECA, where flow characteristics are expected to converge. These observations suggest that the classifier internally models arterial appearances along a spectrum of ICA-CCA-ECA morphology.

The two largest models, AlexNet and VGG16, exhibits smaller focused regions in their GradCAM visualizations. For the CCA and ECA classes, these models display strong activation at the systolic peak, and the late diastolic region. This suggests these models might be internally extracting features resembling a resistance index. Conversely, for the ICA class, the diastolic flow region near the zero axis is highlighted, potentially indicating quantification of diastolic flow volume. The CCA class once again demonstrates a focus on the diastolic notch.

#### LIME

**Fig. 5:**
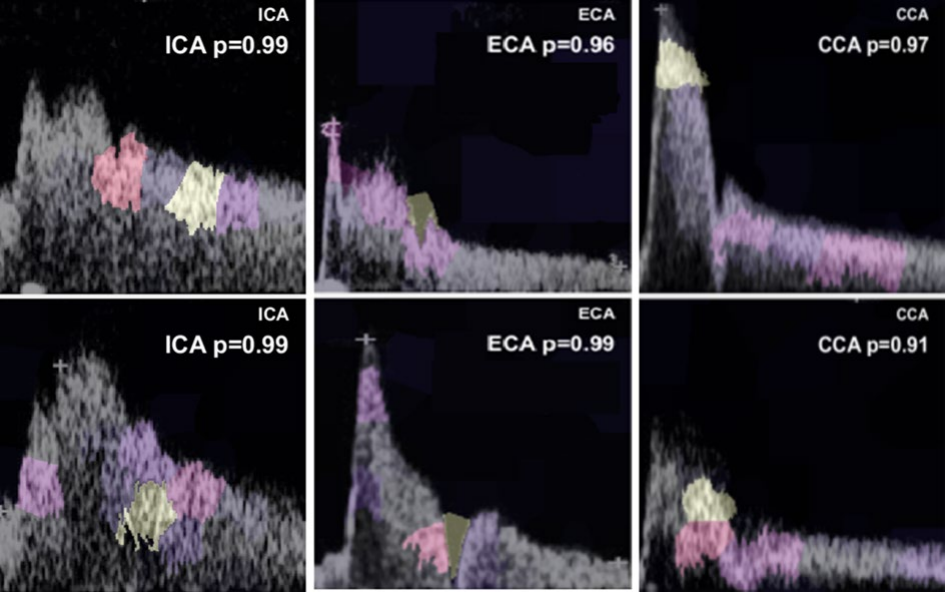
LIME superpixel overlays highlighting features typical of each artery class

LIME analysis generates an image overlay where hyperpixels are colored according to their importance for the model’s prediction. A highly important hyperpixel signifies that removing, adding, or significantly altering that image region would influence the model’s output, potentially leading to a different class prediction. As illustrated in Fig. 5, the LIME output for the ECA class highlights a prominent hyperpixel within the diastolic notch. This suggests that if this gap is filled, the model’s prediction might shift towards the CCA class, for example. The ICA class, consistent with other analyses, displays high importance in the diastolic flow region. The CCA class exhibits activations encompassing both the systolic peak and the diastolic flow region.

### Robustness to noise & artefacts

**Fig. 6:**
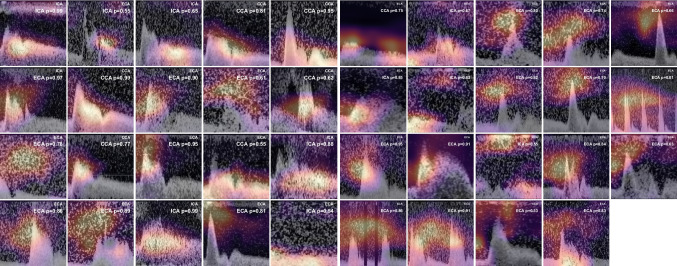
Examples of classifications on very noisy and atypical samples, such as incorrectly segmented beats, aliased Doppler, shifted peaks and multiple peaks. 29 of the 39 samples above (74%) were still correctly classified. On the top right of each image is the true label and below it is the predicted label with confidence score (p)

The deep learning models exhibit resilience to high levels of background noise, provided the underlying signal remained discernible. As shown in Fig. 6, their activations remain focused on the signal region, unaffected by spurious activations from background noise. Conversely, machine learning models heavily rely on robust envelope detection and feature extraction algorithms. The presence of noise hinder their performance, as accurate envelope extraction becomes unreliable. It is noteworthy that outliers and noisy samples are excluded from the machine learning models due to pre-processing failures, whereas noisy samples are included for deep learning. This only further emphasizes the superior performance of the deep learning models. Additionally, deep learning models demonstrate robustness to horizontally shifted samples, a potential consequence of inaccurate envelope or peak/trough estimation.

By incorporating noisy, shifted, and imperfectly segmented samples, the deep learning models inherently learn to be robust to such imperfections. This exemplifies a key advantage of deep learning: feature extraction becomes an integrated part of the model, and data pre-processing requirements are significantly reduced due to the model’s inherent ability to handle noise and artifacts.

### Future work and real-world deployment

In a real-world scenario, carotid Doppler examinations may be performed by non-expert operators using handheld or low-cost spectral Doppler devices. The operator would be trained in the use of the system and on maneuvering the probe as per a predetermined protocol, such as beginning above the clavicle and then moving superiorly up the neck. However, without anatomical visualization or specialist experience, probe placement could be suboptimal, potentially insonifying multiple vessels simultaneously or missing the target artery. A software application that analyzes the Doppler signal in real time could mitigate this challenge: the trained model could continuously assess the incoming waveform and provide the operator with a running weighted prediction of which carotid segment is currently insonified, accompanied by a visual representation of confidence, such as a color scale. The operator could then adjust probe placement to acquire a good quality waveform of the target artery. Simultaneously, a disease-prediction model could analyze the identified artery for cardiovascular abnormalities. A printed report may include a GradCAM analysis to aid clinical interpretability and reasoning.

Future work will include model testing and potential re-training on signals from handheld or low-cost devices, operated by non-experts in a practical setting such as the one described above, to assess real-world generalizability. Focus will be placed on automated classification of ICA Doppler spectra for CVD detection. Potential subgroups of disease could be considered such as degrees of stenosis and overlying cardiac conditions. With larger datasets, the use of vision transformers and attention-based methods could be explored, which are recent advancements capable of deeper feature extraction and accuracy [31]. Including waveforms from various ultrasound systems would improve model generalizability.

While the models have been evaluated on individual beats, the overall performance lies in the classification of an entire sample consisting of multiple beats. The final classification for a waveform could combine the probabilities from each beat, in a voting mechanism, to make the result more reliable.

For practical implementation into a user application, the quantization, compression and optimization of the models for deployment need to be considered for real-time inference and efficient clinical workflow. The small size and fast inference of the GoogLeNet model makes it suitable for low-resource computing.

## Conclusion

Spectral Doppler systems present a unique context and diagnostic challenge due to the lack of anatomical visualization and absolute velocity measurements. While carotid arterial segment identification can be readily achieved with traditional ultrasound, the blind nature of spectral Doppler makes this process error-prone and virtually unachievable in a non-specialty environment. While current clinical guidelines for carotid artery disease focus on indices such as peak systolic velocity (PSV) and end-diastolic velocity (EDV) in the ICA, our approach provides a foundational framework for enhancing the reliability of spectral Doppler systems in resource-limited settings, particularly when operated by non-specialists.

This work has shown that intrinsic patterns in the Doppler spectra can be used by machine- and deep learning methods to accurately distinguish between the common, internal, and external carotid arteries (CCA, ICA, and ECA). This classification is foundational for spectral Doppler to function effectively in a clinical setting without anatomical visualization available. The methods and overall framework presented are suitable for application to Doppler ultrasound spectra in general, including classifying Doppler spectra in other areas of the body, as well as a workflow for multiclass disease detection.

A clinical study enrolled 398 participants who underwent carotid Doppler examination. While data were acquired using a high-end duplex system to ensure high-quality reference data, the trained models operate solely on Doppler spectral images, a format identical to that produced by low-cost spectral Doppler systems. The Doppler waveforms underwent pre-processing and feature extraction. Several classifiers were applied and evaluated, including five well-known deep convolutional neural networks (CNNs) utilizing transfer learning applied to Doppler spectral images, and machine learning classifiers applied to the maximum frequency envelope and extracted Doppler features.

The best performing classifier was the GoogLeNet CNN, achieving a mean area under the curve (AUC) of 0.929 and F1-scores of 0.830, 0.803 and 0.764 for the ICA, ECA, and CCA, respectively. Deep learning models generally outperform traditional machine learning approaches[31], as was also evident during this study. Fine-tuning a pre-trained model offered a performance increase by retaining low-middle level features learnt on a large-scale dataset. CNN-based models also displayed higher noise resilience, likely due to capturing significantly more complex spectral information.

The machine learning models were useful for establishing baseline accuracy and understanding the fundamental features and limitations of the problem. The increased accuracy gained using deep learning suggests that human-perceptible features may not be adequate for attaining ultimate accuracy, especially for the subtleties present in biomedical signals. The use of explanation tools such as GradCAM and LIME in this study provides a human-interpretable system output. It provides valuable insight into the features that are important for the classification task, thus validating the model outputs. This facilitates debugging and optimization during model development and may provide insight and confidence to users during clinical use. Explainability in machine learning-enabled medical devices is essential for transparency, a key requirement of regulatory frameworks. Future work will focus on validating the approach in a realistic, non-expert setting, extending it to disease detection and optimizing models for practical, low-resource deployment.

Overall, integrated AI tools can work for, and alongside, clinicians in an under-resourced environment where there is a lack of cardiovascular specialty. The simplicity and cost-effectiveness of spectral Doppler ultrasound make it a good tool for cardiovascular screening in primary healthcare settings, supporting its widespread adoption and utility in improving healthcare outcomes.

## Data Availability

The data used and generated is confidential.

## Abbreviations

AI: Artificial Intelligence
ASCVD: Atherosclerotic cardiovascular disease
AUC: Area Under the ROC Curve
CNN: Convolutional Neural Network
CCA: Common Carotid Artery
CVD: Cardiovascular Disease
DVT: Deep Vein Thrombosis
ECA: External Carotid Artery
EDV: End-Diastolic Velocity
GradCAM: Gradient-weighted Class Activation Mapping
ICA: Internal Carotid Artery
IMT: Intima-Media Thickness
LIME: Local Interpretable Model-agnostic Explanations
LMICs: Low- and Middle-Income Countries
NCD: Noncommunicable Disease
PAD: Peripheral Arterial Disease
PI: Pulsatility Index
PSV: Peak Systolic Velocity
RI: Resistance Index
ROC: Receiver Operating Characteristic
SNR: Signal-to-Noise Ratio
WHO: World Health Organization
